# Prevalence of Candida Species in Erosive Oral Lichen Planus

**DOI:** 10.5681/joddd.2010.004

**Published:** 2010-03-14

**Authors:** Masoumeh Mehdipour, Ali Taghavi Zenouz, Somayeh Hekmatfar, Mohammad Adibpour, Aila Bahramian, Reza Khorshidi

**Affiliations:** ^1^ Assistant Professor, Department of Oral Medicine, Faculty of Dentistry, Tabriz University of Medical Sciences, Tabriz, Iran; ^2^ Post-graduate Student, Department of Pediatric Dentistry, Faculty of Dentistry, Shiraz University of Medical Sciences, Shiraz, Iran; ^3^ Lecturer, Department of Parasitology, Faculty of Medicine, Tabriz University of Medical Sciences, Tabriz, Iran; ^4^ Post-graduate Student, Department of Pediatric Dentistry, Faculty of Dentistry, Shiraz University of Medical Sciences, Shiraz, Iran; ^5^ Department of Oral & Maxillofacial Surgery, Faculty of Dentistry, Tabriz University of Medical Sciences, Tabriz, Iran

**Keywords:** Candida, erosive lichen planus, etiology

## Abstract

**Background and aims:**

The clinical management of oral lichen planus poses considerable difficulties to the clinician. In recent years, researchers have focused on the presence of pathogenic microorganisms such as Candida albicans in the patients with refractory lichen planus. The aim of the present study was to investigate the prevalence of candida species in the erosive oral lichen planus lesions.

**Materials and methods:**

Twenty-one patients with erosive oral lichen planus and twenty-one healthy individuals aged 18-60 were randomly selected; samples were taken from the tongue, saliva and buccal mucosa with swab friction. Theses samples were sent to the laboratory for determining the presence of candida species in cultures and direct examination method.

**Results:**

No significant difference was found between healthy individuals and patients with erosive lichen planus regard-ing presence of candida species. The type of candida in the evaluated samples was Candida albicans in both healthy and patient groups.

**Conclusion:**

According to the results, candida was not confirmed as an etiologic factor for erosive lichen planus lesions.

## Introduction

Oral lichen planus (OLP) is a chronic inflammatory oral mucosal disease with an unknown etiology. The clinical management of OLP poses considerable difficulties to the clinician. Although the etiology is unknown, most researchers believe that immunologic factors are involved.^1^ World Health Organization introduced this disease as a complication, which increases the risk of cancer.^2^ Although immune system disorders are considered as common etiologic factors involved in lichen planus, several treatment modalities have been proposed, each with pertinent side effects.^3^ In recent years researchers have focused on the presence of pathogenic microorganisms, such as Candida albicans in patients with leukoplakia and refractory lichen planus. C. albicans is present in about 37% of OLP lesions.^5 , 6^ Symptoms of OLP may be exacerbated by candida overgrowth or infection, while antifungal treatment of erosive lesions with candida can transform the lesions into the reticular form.^7 , 8^ Theoretically, the use of antifungal agents in some cases of OLP can reduce the potential of C. albicans to produce carcinogenic N nitrosobenzylmethylamine.^9^ It is difficult to establish whether there is a synergistic pre-malignant effect in case of exposure to potentially carcinogenic substances (contributing external risk factors) and persistence of OLP (intrinsic risk factor).

Candida infection has been shown to be present in 17.4% and 16.4% of ulcerated and non-ulcerated OLP cases, respectively.^6^ Some C. albicans isolates with special genotypic profiles and virulence attributes might contribute to the development and progression of OLP.^10^ In fact, C. albicans might play different roles in different mechanisms in two groups of patients, including oral pre-malignant lesions and oral cancers.^11^ The purpose of the present study was to determine prevalence of candida species and to evaluate whether specific types of candida are associatedwith OLP lesions or not.

## Materials and methods

This descriptive, cross-sectional case-control study included individuals with lichen planus referred to the Department of Oral Medicine at Faculty of Dentistry, Tabriz University of Medical Sciences. Twenty-one patients, 18-60 years old, with erosive oral lichen planus as the case group and 21 healthy individuals, 18-60 years old, without symptoms of disease or any infections, as the control group, were selected.

A card containing the following data was designed for each individual patient: 1) file number, age, sex, occupation and duration of OLP (history) until clinical examination; 2) clinical picture of lichen planus with the pertinent form (erosive, bullous) and presence of C. albicans; 3) history of systemic diseases such as diabetes mellitus, presence of any predisposing factors for the incidence of candidiasis such as long-term consumption of antibiotics and corticosteroids, and tobacco use.

The diagnosis of C. albicans infection was based on the findings from cultures of samples, taken with an oral mucosal swab from saliva as well as tongue, and buccal mucosa, and transferred to Sabaurad’s Dextrose Agar (SDA) and Sabaurad plus chloramphenicol (SC) media at room temperature (20°C).

The sample swabs were expanded on two other clean slides and stained for direct examination by 10% potash and methylene blue. Expanded samples were evaluated for the presence of yeast cells and pseudomycelium. Yeast cell numbers more than normal or with pseudomycelium were diagnosed as candidiasis. Yeast cells have a rapid growth within 2-3 days in vitro, and generate cream yeast colonies. A suspension was prepared from the colonies on the clean slide next to a flame inside a drop of sterile saline and then was evaluated under a microscope for confirmation of yeast cells and mycelium. If the yeast cells and mycelium were present, to confirm C. albicans and several common species of candida two methods were used:

1) Production of germ tube by C. albicans: More than 90% of the Candida albicans microorganisms create germ tube inside the new human serum or fetal bovine serum at a temperature of 37°C for 2-3 hours. Some new yeast colonies were removed by fildoplatin to carry out this test, which were solved in 1 mL of fresh human serum in sterile test tubes. C. albicans was reported if there were germ tubes after 2-3 hours in a drop of suspension under a microscope.

2) Production of color in color field: An environment with the property to produce different colors has the ability to determine C. albicans, Candida tropicalis, Candida krusei and Candida glabrata. New yeast colonies grown were transferred to SDA and SC environments in chrome agar culture medium to carry out this test. After 48 hours, we evaluated the color produced. In this culture medium C. albicans creates green color, C. tropicalis creates blue with a metallic appearance, C. krusei creates light red and C. glabrata creates dark red.

Data were analyzed with Fisher’s exact test using SPSS 13.0 software.

## Results

There were 20 female (47.6%) and 22 (52.4%) male patients. The patients’ age range was 18-60 years. In investigating the samples sent to the laboratory for direct examination method, yeast cells with higher than normal counts or with pseudo-mycelium were diagnosed as candidiasis. There was no significant difference in the presence of candida species between the two groups (P = 0.36). Furthermore, comparison of the samples in the culture medium did not demonstrate any significant differences between the two groups (P = 0.25) (Table 1). To describe the type of candida in both groups we used two methods: (1) production of germ tube; and (2) production of color in the color field. In this study in both groups all the samples with positive cultures were able to produce breeding tube except for a case observed in healthy individuals. All the samples prepared from both groups produced green color in chrome agar culture medium except for one case in the control group that indicated C. krusei. This result was similar to that of the previous test due to the inability to produce germ tube.

**Table 1:** Prevalence of candida species in direct examination and culture medium, and Candida albicans in germ tube between case and control groups

Group	Direct examination	Medium culture	Germ tube
Case	%33.3	%33.3	%33.3
Control	%23.8	%28.5	%19.04

## Discussion

The aim of this study was to investigate the prevalence of candida species and the common species in erosive OLP. The results of the present study did not demonstrate any significant differences between healthy individuals and patients with erosive lichen planus regarding candida presence, but C. albicans was the dominating species.

The results of the present study are consistent with the results of a previous study, which reported that candida infection occurs more readily in OLP and non-specific lichenoid stomatitis (NSLS), with no apparent association with ulceration in OLP.^6^ The comparatively marked increase in the infection prevalence of ulcerated NSLS was not statistically confirmed, and its significance remains uncertain.^6^

OLP has considerably less susceptibility than oral leukoplakia to invasion by C. albicans.^12^ It has been suggested that further evidence is provided supporting the hypothesis that certain strains of C. albicans and yeasts play an etiologic role in the development of oral cancer by means of endogenous nitrosamine production.^13^ One study showed that presence of candida was significantly correlated to low secretion rate of unstimulated saliva.^14^ These results might be attributed to different samples or to the use of different scoring methods, which make comparison of the results difficult.

## Conclusion

Although the prevalence of candida in OLP patients did not exhibit a statistically significant difference compared to healthy subjects, the current type of candida was C. albicans in both healthy and unhealthy groups. Identification of species responsible for infection is important for successful clinical management and for determining appropriate control measures to prevent transmission of resistant candidal pathogens. However, further studies are necessary to substantiate such relationship.
